# Engaging with holism in Australian Aboriginal health policy – a review

**DOI:** 10.1186/1743-8462-2-15

**Published:** 2005-07-13

**Authors:** Mark Lutschini

**Affiliations:** 1 Centre for the Study of Health and Society & VicHealth Koori Health Research and Community Development Unit, Department of Public Health, University of Melbourne, Melbourne, Australia.

## Abstract

**Background:**

The ideal concept of Aboriginal holistic health is centrally placed in Australian Aboriginal health policies and strategies. Its effective uptake promises, as advocates suggest, reorienting the complex Australian health system to enable health improvements. However, continual reminders assail us that Aboriginal health is shocking, appalling, disastrous, disgraceful and damning. Could incapacity to engage effectively with the concept undermine health system improvements? The aim of this review of Australian literature was to identify the range of meanings attached to Aboriginal holistic health and engage with their implications for the health system.

**Results:**

In terms of literature synthesis I found that policy makers cannot rely on this approach to provide coherent arguments for meaningful engagement with the concept because authors in general: are uncritical and un-reflexive in the use and interpretation of the concept; often provide no reference for their understandings; tend to alter the concept's definition and constituent elements without justification; ignore the wide range of mainstream literature about holism and health; and fail to acknowledge and examine the range of Aboriginal concepts of health. I used the ten themes from this literature to highlight implications for the health system, and found that a most profound contradiction exists in the acceptance of the English language concept 'holistic' as immutably Aboriginal. Additionally, a range of contradictions and mixed messages within the themes challenge the validity of the concept. Significantly, with the boundary of the concept constructed as diffuse and ethereal, the diverse and uncritical literature, and mixed thematic meanings, it is possible to justify any claim about the health system as holistic.

**Conclusion:**

It seems not so much incapacity to engage, but incapacity to coherently articulate Aboriginal concepts of health, which prevents advisory bodies such as the National Indigenous Council to imbue whole-of-government approaches in accordance with Aboriginal values.

## Background

The seemingly entrenched poor health status of Australian Aboriginal peoples is for the Commonwealth Government 'a glaring and intractable problem' [1:90]. Since the 1970s, the concept of holism is advocated as providing 'a new way of thinking in Aboriginal health' [2]. The concept is often acknowledged as the Aboriginal definition of health (hereafter the Definition):

Health is not just the physical well-being of the individual, but the social, emotional, and cultural well-being of the whole community. This is a whole-of-life view and it also includes the cyclical concept of life-death-life [3:ix]

However, beyond the Definition there is little detail as to the general and specific effects for the health system that acceptance of the concept implies. Therefore, in order to develop an improved knowledge base, a standard approach for policy makers is to perform or commission a literature review on a topic, and then distil the key themes and related meanings. Thus, a greater understanding may be achieved to enable more effective transfer of meanings into the complex Australian health policy environment. The genesis of this study began with a question often faced by policy makers: what is the holistic concept of Aboriginal health and how does it affect health policies, strategies, and programs? From a review of Australian Aboriginal health literature emerged ten themes that I use as focal points to highlight a number of conflicts and tensions complicating policy makers' effective engagement of Aboriginal holistic health.

## Results

### Literature Characteristics

Table 1 shows the diversity of the 153 publications detected (structure), with journal articles (28%) and reports (31%) constituting the bulk of publications (59%). In terms of publication origin, a total of 39% came from governments compared to 16% from Aboriginal organisations, followed by 15% from 'other' – publications with no explicit organisational affiliation. In terms of year of publication, the release of the National Aboriginal Health Strategy in 1989 is followed by a steady and rapid increase to peak at 74 publications in the period 2000–2002. The citation source is the explicit reference by the authors of their source of holistic, with 24% referring to the NAHS, 12% to 'other' sources such as Aboriginal reports and research, and 64% providing no source. The Definition was directly cited as the source of holistic in 12% of the publications.

**Table 1:** Characteristics of the Health Professional Literature*

**Structure**	**n**	**%**	**Origin**	**n**	**%**
Book	6	4	Professional Association	5	3
Policy	9	6	Research Centre	10	7
Monograph	10	7	Multi-institutional	15	10
Inquiry Submission	10	7	University	16	10
Health Strategy	18	12	Other	22	15
Journal Article	43	28	Aboriginal Organisation	24	16
Report	48	31	State Government	25	16
			Commonwealth Government	35	23

***Total***	***153***	***100***	***Total***	***153***	***100***

**Year**	**n**	**%**	**Citation Source**	**n**	**%**

1988 – 1990	1	1	NAHS	36	24
1991 – 1993	6	4	Other	19	12
1994 – 1996	19	12	None	98	64
1997 – 1999	36	24	***Total***	***153***	***100***
2000 – 2002	74	48			
2003 – 2004	14	9	NAHS Definition	18	12
Unknown	3	2			

***Total***	***153***	***100***			

*n = number; % = percent of total; citation source refers to the referenced source of holistic.

### Content and Thematic Analysis

Table 2 shows the themes of Aboriginal holistic health. The number of times a theme occurred in the literature, as evident in the examples, are counted (n) and presented in ascending order.

**Table 2:** Thematic Boundaries Constructing Aboriginal Holistic Health*

**Theme**	***n***
**Problems with the Aboriginal holism** Examples: difficult to define; holistic health care compounds unrealistic expectations; holistic concept is used to distract health services from their core business; and data definitions and standards not adequately developed to encompass holistic view.	**9**
**Concept Confusion** Examples: ecological model, WHO definition of health, primary health care, ethnomedicine, and social medicine.	**17**
**Consistent with Comprehensive Primary Health Care** Examples: holistic comprehensive primary health care; CPHC is holistic; supports provision of CPHC; and holistic CPHC services.	**26**
**Essential to Improved Health Status** Examples: it must be understood that when the harmony of these interrelations is disrupted, Aboriginal ill health will persist; improvement of Aboriginal health depends upon more holistic systems; and a holistic approach to the delivery of services is essential to the improvement of Aboriginal health.	**27**
**Opposite of the Western, Biomedical Approach** Examples: holistic lifestyle opposite of European lifestyle; not built around specialities or body parts; in contrast to mind/body dichotomy of biomedicine; and body parts programs conflict with principle of holistic health.	**29**
**Exemplified in Aboriginal Community Controlled Health Services** Examples: Indigenous services insist on an holistic understanding; Aboriginal medical services incorporate an holistic approach; Aboriginal community controlled health services take account of the holistic context of service delivery; and they deliver holistic primary health care.	**38**
**Mainstream Health System Failure** Examples: fragmentation of roles; lack of coordination; areas that affect health outside the health portfolio; and vertical and inflexible programs.	**39**
**Broad View of Health** Examples: broader context of health; whole of life cycle; multi-faceted view of health; and encompass all aspects of life.	**42**
**Embodied by Aboriginal People** Examples: the holistic view of health traditionally held by Indigenous people; Aboriginal concepts of health are holistic; acceptance of Aboriginal peoples' holistic view of health; and a holistic Aboriginal concept of health.	**59**
**Underpinning Philosophy of Health** Examples: Aboriginal holism should be an underlying principle and philosophy of policy, program development, service delivery, strategies, and practice.	**75**

*n = number of instances a theme was evident in statements

### Elements of Aboriginal Holistic Health

Table 3 shows the number of times (n) that authors stated constituent elements of the concept. The elements from the Definition were overall the most commonly stated (italicised), with other elements included (non-italicised) at a lower number.

**Table 3:** Inter-related Elements of Aboriginal Holistic Health*

Element	n	Element	n
Ideological	2	Economic	9
Lifestyle	2	Mental	9
Nutrition	2	Physical Environment/Infrastructure	11
Service Environment/access	2	*Individual*	25
Education	3	Spiritual	29
Governance	3	*Well being*	29
Identity	3	*Community *(development, capacity, leadership)	32
*Life-death-life*	5	*Emotional*	33
Land	6	*Physical*	33
Political Environment	6	*Cultural*	35
Whole-of-life-view	6	*Social*	38
Family	7		

*n = number of instances an element was stated

## Discussion

There is no definitive source providing a comprehensive grounding framework to enable effective engagement with the concept of Aboriginal holistic health. Therefore, policy makers have to navigate and interpret a diverse health literature and assemble disparate messages into saleable policy options. In this discussion, I attempt to show how an examination of this literature raises a number of tensions underlying attempts to meaningfully transfer Aboriginal cultural concepts into health policy. This was a text-based study as access to and use of easily accessible published literature is a prime tool in a policy maker's kit. However, it is important to note that the study findings would be improved through interviews and the inclusion of unpublished literature and transcripts of speeches and presentations. Nevertheless, the outcomes of literature reviews are often the first point in establishing the conceptual framework of health policy, strategies and programs.

### Immutably Aboriginal?

The literature conveys a strong sentiment that holism is embodied by Aboriginal people (Table 2), and therefore it was necessary to find and understand its original source. The Definition was apparently first written in 1974 by the National Aboriginal and Islander Health Organisation (NAIHO, now the National Aboriginal Community Controlled Health Organisation, NACCHO), however I could not obtain Beaton's citation [5]. The 1989 National Aboriginal Health Strategy (NAHS) appeared as the next likely source. Since the release of the NAHS the explicit use of 'Aboriginal holistic health' in publications increased from 6 references in the period 1991–1993 to peak at 74 references during 2000–2002 (see citation source, Table 1).

In 24% of these publications, authors explicitly cite the NAHS as their source of holistic health (Table 1), in which it is mentioned twice – first on page 60 and the second much deeper in the document:

The Working Party has endorsed the need for a wholistic approach to improving Aboriginal health. This approach will encompass social, cultural, political, economic, environmental and physical factors, not all of which are easy to quantify. [3:219]

However, the NAHS did not provide a source for 'holistic', a practice repeated in 64% of the publications (Table 1). Additionally, the NAHS does not explicitly link 'holistic' and the Definition (which occurs on page x), although 12% of publications did (Table 1). Therefore, within the Aboriginal health literature it is unclear from where 'holism' originated.

Furthermore, no publication in the Aboriginal health literature referred to the extensive non-Aboriginal literature on holistic health. Holism occurs in mainstream health documents such as the National Health Priority Action Areas [6], and the Victorian government's Municipal Public Health Planning Framework [7]. It is a popular keyword in the Australian Journal of Holistic Nursing; is a philosophy extending into the realm of medical doctors as 'alternative' or 'complementary' medicine [8-11]; and is advocated as underlying a 'new kind of GP' [12]. Neither these strategies nor two reviews of holism and health in the non-Aboriginal literature referenced or discussed Aboriginal perspectives [8,13].

Among the five 'reviews' about Aboriginal understandings of health, when using 'holistic' none investigated the root of the word [14-18]. This uncritical acceptance is mirrored in the only research project based on the concept [19,20]. However, the Oxford English Dictionary and the Barnhart Dictionary of Etymology stated that the terms holism and holistic were coined in 1926 by the biologist and former South African Prime Minister Jan Christiaan Smuts [21-23]. Their definitions have obvious connections with the Definition, where both invoke the interconnectedness or 'whole' aspects of holism:

'parts of a whole are in intimate interconnection, such that they cannot exist independently of the whole, or cannot be understood without reference to the whole, which is thus regarded as greater than the sum of its parts' [22:828]

'to designate the tendency in nature to produce wholes (i.e. bodies or organisms) from the ordered grouping of unit structures' [23:307]

It could be surmised that the appropriation of an English language construct was necessary as it is claimed that:

'... while Aboriginal languages do accommodate the complex inter-related constructs involved, Western languages cannot and nor can the relevant Aboriginal constructs be translated' [24:90]

Perhaps this partly explains why the concepts of *mwarre*, *punyu*, and *wankaru *[15,25,26] do not receive any in-depth attention. Additionally, the cross-cutting rivalries and intra-cultural conflict within Aboriginal Australia could be another reason, as the advocacy of one term from one Aboriginal group is seen as an offence to other groups. It also seems difficult to believe that after thirty years of existence of the concept, Aboriginal health leaders and health professionals could not better articulate its meanings, or the meanings of other concepts. An understanding of the meanings attached to these concepts would prove valuable to policy makers who are advised to consider the heterogeneity of Aboriginal cultures [27]. This has significant implications for consultation mechanisms, as outline further below.

Finally, a profound contradiction exists in that 'holism' in text originates from a Western source, but the NAHS states that 'Aboriginal culture is the very antithesis of Western ideology' [3:ix]. Furthermore, although exclusively written in the English language the NAHS is referred to as an 'Aboriginal document' [28]. If culturally based concepts allude to a range of meanings, and if 'such meanings delineate the conceptual field within which action develops' [26:65], then whose cultural base is it?

### Essential to Improve Health?

The uncertainty about holism's 'Aboriginality' disempowers the idea that it is essential to improve health (Table 2). Adding to this is the variation of wording and constituent elements of the Definition. For example, NACCHO use this version in their 2003–2006 business plan:

Aboriginal health is holistic, encompassing mental health and physical, cultural and spiritual health. Land is central to well being. Crucially, it must be understood that when the harmony of these interrelations is disrupted, Aboriginal ill health will persist. [29:5]

This significantly diverges from the Definition – where did the social and community go and how did land become central? To what specific interrelations do they refer? In trying to clarify the interrelated elements, in the literature most of the time authors replicated elements stated in the Definition (Table 3), while other elements are added, subtracted or modified without a justification for doing so. The Definition is frequently inserted into documents in cut-down, re-worded and re-phrased versions. This is from the *National Aboriginal and Torres Strait Islander Health Strategy: Framework for Action by Governments *(hereafter the Framework):

A holistic approach: recognising that the improvement of Aboriginal and Torres Strait Islander health status must include attention to physical, spiritual, cultural, emotional and social well-being, community capacity and governance. [27:2]

The Framework was developed by the National Aboriginal and Torres Strait Islander Health Council, agreed to by all governments, and critically, it claims Aboriginal ownership through extensive consultations. This places the concept in a central strategic position to frame actions in the health system. However, how can it be given due merit considering the implications about its cultural validity in combination with a selective use of constituent elements and shifting definitional boundaries?

### Mixed Messages

Furthermore, in considering the integrity of the publications, they come from a wide variety of authors from different disciplines, writing in different styles of publication with various organisational affiliations (Table 1). Their understanding, interpretation and application of holism when they write about Aboriginal health are questionable. This is evident in the mixed messages of confusion, opposition, connection and alignment between Aboriginal and Western ideas of health.

There is conceptual confusion (Table 2) where some authors explicitly link holism to the World Health Organization's definition of health, an ecological or ecosystems approach, a new public health approach, and a systems model of thinking [30-37]. The working party of the NAHS quoted the WHO definition of primary health care in full [3:x], without questioning its cultural origins. Additionally, the Framework states that:

Within the health system, the crucial mechanism for improving Aboriginal and Torres Strait Islander health is the availability of comprehensive primary health care services. [27:1]

The acceptance and uptake of this Western approach appears unproblematic in the NAHS and the literature where Aboriginal holistic health is consistent with comprehensive primary health care (Table 2). Further confusion ensues through the conceptual overlap with primary care, comprehensive primary health care [38,39], and vertical primary health care, which 'forms only a part of comprehensive primary health care, which is a broader, holistic approach to health problems' [40:146]. These differences appear trivial, but their philosophical underpinnings have important implications for operationalisation into health system [38,39].

Both the mainstream and Aboriginal health literature position holism as opposite, counter, or antithetical to the Western biomedical model of health [8,13,26,41-45]. Three senses to this discourse emerge from the literature, the most prominent being the health professions past ethos of colonialism – 'the domination of Aboriginal health care by the medical model approach fitted well with other assimilation policies of the period' [3:59]. In a second sense biomedicine, in contrast with holism, is said to devalue or disregard personal and societal social, economic, environmental and cultural factors on health [33,46]. A third sense relates to biomedicine's Enlightenment rationalist heritage which tends to emphasise a mechanistic view of the body, reducing 'health' to an absence of biochemical and physiological symptoms [33,47].

However, there seems to be a degree of connection between biomedicine and holism because the phrase 'not just the physical well-being of the individual' of the Definition seems to connect with biomedical constructions. Additionally, both the NAHS and the Framework heavily depend on biomedical indices (statistics of illness) in the construction of health need. Therefore, there are points of connection between Aboriginal concepts of health or well-being and biomedical knowledge, as Anderson has noted [26].

Furthermore, there seems to be some alignment between Western and Aboriginal views as both accept the need for a broad view of health. The 1948 WHO definition of health underpins the 1978 Declaration of Alma Ata as accepted in Australian through the 1979 'Health for All' policy [15]. These developments precede the NAHS (1989), and there are similarities between the Definition and the WHO version of health. The Australian health system is also more accepting of other world views in what Heather Eastwood (2000) calls a 'postmodernist movement' that challenges the superiority of the positivist, modern world view. Also, policy makers are more aware of the need to consider ethnicity and culture in health policy [48].

These confusing messages detract from the clarity needed to support the theme of Aboriginal holism as an underpinning philosophy of health (Table 2). What they also signal is that both Western and Aboriginal societies are heterogeneous and contain a multiplicity of health concepts [26,49]. Moving the discussion from a conceptual level to the service delivery level, the literature contains further contradictory meanings.

### Which Health System Failure?

Aboriginal people access both Aboriginal-specific and mainstream health services [38,50]. This contrasts with a theme suggesting the failure of the mainstream health system to address Aboriginal health (Table 2), principally because health 'is holistic, a concept that many Western models of healthcare delivery fail to identify and therefore accommodate' [42:222]. Nevertheless, some advocates are fervent that mainstream services should [51], and have [52] become more holistic. Therefore, it is no surprise that the Commonwealth Health portfolio:

is pursuing a two pronged approach, which aims to both improve accessibility and responsiveness of the mainstream health system and to provide complementary action through Indigenous specific health programs. [53:161]

The efficacy of this policy seems justifiable as large potential gains in Aboriginal health could be realised through reorienting Australia's $66.6 billion health system [54]. Advocates readily support work to improve mainstream system access following from many initiatives developed in the NAHS and the Framework. Additionally, governments fund and support more than 100 Aboriginal community controlled health services (ACCHS) initiated since 1971 [24,50] that are said to exemplify holism (Table 2). Published literature from ACCHS would assist policy makers in clarifying service delivery implications of holism, however only 16% of the publications came from Aboriginal organisations (Table 1).

Some descriptive literature is available [55,56] but none based on evaluations of their relative effectiveness [57], although community controlled service delivery is often uncritically proclaimed as 'an uncontested good' [57:4]. Furthermore there is an array of different types of Aboriginal service delivery models covered under the banner of holistic services, ranging from dedicated Aboriginal medical services to multi-purpose cooperatives with health as part of their overall function, to separate substance misuse services [56]. This lack of critical evaluation research is a major impediment to understanding the efficacy of ACCHS, and how the lessons of their operations could influence mainstream health system reforms.

This adds to the theme where some authors note problems with the concept (see Table 2), which include that relying on it may divert attention and resources away from the core business of health services, and lead to difficulties in establishing effective measures and indicators [49,58,59]. However, they fail to elucidate specific criteria and evidence for their assertions. These factors combine with the findings outlined above mean any argument for success of failure of the health system has limited empirical basis for justification. As such, the Commonwealth could justify saying that:

The health needs of Indigenous Australians are largely met through the funding and delivery of mainstream health services, with services specially targeting Aboriginal and Torres Strait Islander people complementing these mainstream services. [60:v]

In a broader sense, often accused of poor coordination Commonwealth Government agencies are beginning 'to engage in holistic thinking and think beyond the boundaries, conceptualise broad outcomes, and understand areas of commonality' [61:53]. However, government officials face a maze of uncoordinated Indigenous structures to navigate [62]. Of the thousands of Aboriginal organisations the potential exists for an 'Indigenous order of Australian government' [63], but attempts such as coalitions of Aboriginal organisations [64,65] fail to present coherent policy positions.

### Health Professional Separatism?

In terms of health service delivery, authors are quite willing to claim that Aboriginal people embody holism (Table 2). For example, within ACCHS 'the Health Worker role is driven by a holistic approach' [66:xiii]. Furthermore, the Queensland Department of Health ' understands that recruiting Indigenous peoples will better position Queensland Health to... develop a holistic approach to health' [67:3]. These meanings imply a strict separatism between Aboriginal people and ACCHS and non-Aboriginal people and mainstream services.

An implication of this is seen in the definition of community control where 'Aboriginal people must determine and control the pace, shape and manner of change and decision-making at local, regional, state and national levels' [24:77]. Seemingly in agreement, the Australian Medical Association (AMA) supports Aboriginal holism [68]. However, they staunchly defend placing doctors at the centre of primary health care: 'the AMA strongly recommends that all PHC should be delivered through general practice' [69:4] and emphasise 'The unique clinical skills of GPs in providing holistic/social care' [69:2]. Additionally, in spite of the assimilation label attached to the medical profession in the NAHS, the NAHS Working Party accepts the WHO primary health care model developed under the conceptual umbrella of the WHO definition of health. This was developed by experts (professors and medical doctors) with a background in social medicine, one of whom became the first Director-General of the WHO [49].

In practice, mainstream doctors question their own scientific training in providing health care [8], and there is an increased number of both doctors working in Aboriginal health services, and of Aboriginal doctors [50]. Medical doctors receive particular criticism in Aboriginal health, but they have a significant role in the advocacy for Aboriginal rights and in working with Aboriginal organisations [70]. Medical educators have access to medical curricula improvements to incorporate Aboriginal health [71,72]. There also exist a wide range of internet resources specifically targeting the health professional [73-75]. The proposition of separatism between Aboriginal and non-Aboriginal people, concepts of health and antithetical, unchanging cultures appears undercut by the willingness of health professionals to challenge culturally established norms, and to be culturally sensitive in their approach.

### Implications for Health Policy and Strategy Development Processes

I posed the question earlier: could incapacity to engage effectively with the concept undermine health system improvements? I have shown that through the medium of literature synthesis that effective engagement is not possible which is a significant barrier for systemic reform because health strategy construction relies heavily on published literature. This partly undermines the theme of an underpinning philosophy of health (Table 2) as accepted as an explicit policy principle by the Victoria, Northern Territory, New South Wales, Queensland, and Western Australia Governments, and as advocated for as a policy principle by professional associations and a range of non-Government organisations.

However, arguably more meaningful engagement occurs through oral communication. Consultation meetings include a wide range of Aboriginal and non-Aboriginal people across the country. This multitude of voices project preferences through ideological, institutional, professional, and cultural perspectives. While advocates may agree with the concept in principle, each could be tapping into their particular view of holism. For example, some medical literature suggests doctors view 'holistic treatments' as alternative or complementary medicine such as acupuncture, relaxation, massage, and hypnotism [8,41,43].

Additionally, strategies receive ministerial endorsement after processing through a hierarchy of advisory and consultative committees. For example, in the development of the *National Aboriginal and Torres Strait Islander Nutrition Strategy and Action Plan*, the majority-Aboriginal working party was in effect a sub-sub-sub-committee of the endorsing committee: the Australian Health Ministers Advisory Council [76]. This and other endorsing councils do not include an Aboriginal person to explain the complexities and nuances of Aboriginal cultural concepts, and thus revert to understandings from the literature or from their own perspective, clearly as many authors do in the literature.

It could also be argued that one Aboriginal person on endorsing committees would be insufficient, given the hundreds of Aboriginal groups in Australia. This extends to the validity of the majority Aboriginal membership mandate of steering committees, as each may draw on distinct cultural concepts, such as *mwarre*, *punyu*, and *wankaru*. The in-group rivalries are well noted between different Aboriginal tribes as well as between urban, rural and remote communities [77,78]. Finally, the insignificant number of Aboriginal people in all the health system's structures – in 2001 only 0.9% of health care providers were Aboriginal [79:18] – precludes occupying enough positions to give adequate force to oral transfer.

It is perhaps for these reasons that the new reporting framework for the Council of Australian Governments (COAG), set in the *Overcoming Indigenous Disadvantage: Key Indicators *report [80], has a complete absence of references to any Aboriginal concept of health. Significantly, the COAG reporting framework effectively orients all commonwealth ministers to produce efforts consistent with this framework. It is therefore questionable whether Aboriginal concepts and knowledge could receive full argumentation and explanation in these processes, because policy 'is the outcome of the competition between ideas, interests, and ideologies' [81:3]. As such, given the heterogeneity of Aboriginal cultures, ineffective transfer of meanings through written and oral communication, undercut by rivalries and intra-cultural conflict preventing national Aboriginal unification, then the approach of the Howard government of dealing directly with individual Aboriginal communities could well be justified [82].

## Conclusion

I conclude that literature synthesis cannot provide an answer to the question: what is the holistic concept of Aboriginal health? Rather, policy makers can justify any answer based on the diversity of the literature, subsequent themes and range of meanings. Furthermore, I question the cultural basis advocates attribute to the concept as immutably Aboriginal, which undermines the credibility of its use. In terms of the range of Aboriginal concepts available, the effective transformation of their meanings into the health system could be enhanced through coherent and articulate written and oral communication. The lack of knowledge about them is serious barrier that should be addressed through more research. This would, in part, enhance the capacity of policy makers to engage meaningfully and confidently with Aboriginal concepts of health. However, further attention needs to be given to the processes of health policy and strategy development so that these meanings receive adequate consideration and argumentation in written and oral forms. Otherwise, health outcomes for Aboriginal people may continue along an appalling path.

## Methods

### Literature Detection

The Australian Aboriginal health literature was defined as Australian publications on Aboriginal affairs with a health specific focus or section and which explicitly stated the terms 'holistic' or 'wholistic'. Publication detection occurred using the search string: (Aborig* OR Indigenous) AND (holistic OR wholistic) AND health, applied to the databases of *Informit*: APAIS (health, public affairs), ATSIhealth, CINAHL, Health and Society, Australian Medical Index, Rural; as well as MEDLINE and PubMed. Results from each database were cross-referenced and duplicate items removed, with periodic repeat searches ensuring new publication capture. Additionally, snowball searching of journals enabled capture of older publications and those not listed in the databases. Finally, Aboriginal affairs reports were detected through relevant government and organisational internet sites and searched for key terms. The publication characteristics are shown in Table 1.

### Content and Thematic Analysis

Through content analysis, the various statements and phrases explicitly associated with the key terms were entered into a spreadsheet and grouped into common categories to 'count' (Table 2 – n) the number of instances a theme occurred. Content analysis is not concerned with the subject categories' production, reception or with any effects [4], and the count number should not be taken as the sole indicator of the importance or otherwise of a theme. The themes emerged from the overt and implied meanings of statements and evolved with multiple readings of the literature, until a clear list emerged, and at times more than one theme was evident within a statement (Table 2).

## List of Abbreviations

ACCHS – Aboriginal Community Controlled Health Services

AMA – Australian Medical Association

COAG – Council of Australian Governments

GP – General Practitioner

NACCHO – National Aboriginal Community Controlled Health Organisation

NAHS – 1989 National Aboriginal Health Strategy

NAIHO – National Aboriginal and Islander Health Organisation

PHC – Primary Health Care

WHO – World Health Organization

## Competing interests

The author(s) declare that they have no competing interests.

## Authors' contributions

ML is solely responsible for the study.
